# Synthesis and Characterization of Redox-Responsive Disulfide Cross-Linked Polymer Particles for Energy Storage Applications

**DOI:** 10.1021/acsmacrolett.1c00682

**Published:** 2021-12-09

**Authors:** Garrett L. Grocke, Hongyi Zhang, Samuel S. Kopfinger, Shrayesh N. Patel, Stuart J. Rowan

**Affiliations:** † Pritzker School of Molecular Engineering, University of Chicago, Chicago, Illinois 60637, United States; ‡ Joint Center for Energy Storage Research, Argonne National Laboratory, Argonne, Illinois 60439, United States; § Chemical Sciences and Engineering Division, Argonne National Laboratory, Argonne, Illinois 60439, United States; ∥ Department of Chemistry, University of Chicago, Chicago, Illinois 60637, United States

## Abstract

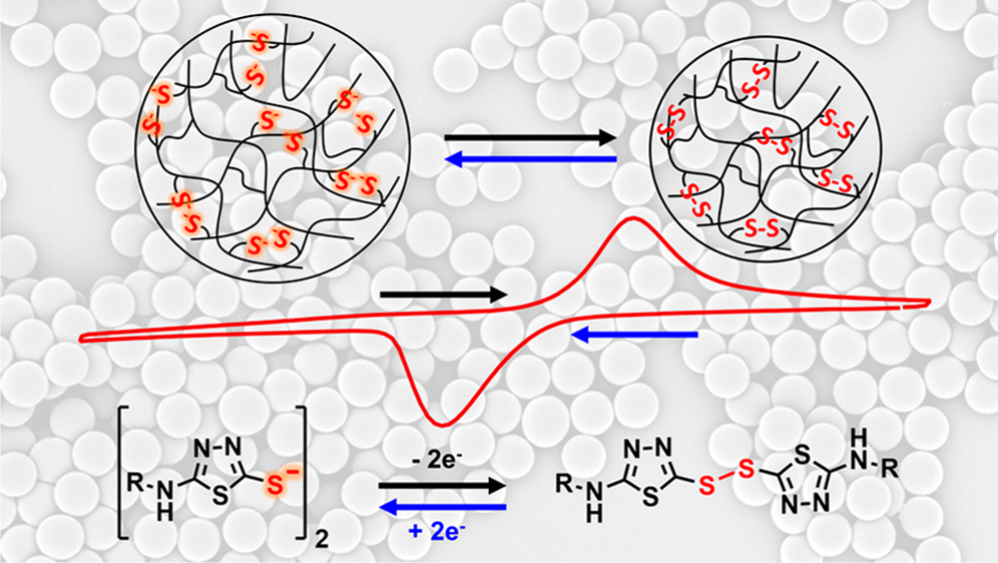

Cross-linking poly(glycidyl methacrylate) microparticles with redox-responsive bis(5-amino-l,3,4-thiadiazol-2-yl) disulfide moieties yield redox-active particles (RAPs) capable of electrochemical energy storage via a reversible 2-electron reduction of the disulfide bond. The resulting RAPs show improved electrochemical reversibility compared to a small-molecule disulfide analogue in solution, attributed to spatial confinement of the polymer-grafted disulfides in the particle. Galvanostatic cycling was used to investigate the impact of electrolyte selection on stability and specific capacity. A dimethyl sulfoxide/magnesium triflate electrolyte was ultimately selected for its favorable electrochemical reversibility and specific capacity. Additionally, the specific capacity showed a strong dependence on particle size where smaller particles yielded higher specific capacity. Overall, these experiments offer a promising direction in designing synthetically facile and electrochemically stable materials for organosulfur-based multielectron energy storage coupled with beyond Li ion systems such as Mg.

The development and use of organic materials that can be employed in the next generation of batteries has seen an increase in interest over the past decade or so. Such interest has been spurred by a number of factors,^1^ such as the design/development of many redox-active organic molecules that offer competitive if not higher theoretical specific capacities than current inorganic electrode offerings.^2^ Furthermore, the ability to systematically tune the structure of organic materials allows fine-tuning of their electrochemical behavior/stability, and the raw materials for organic systems are generally more abundant compared to many of the key metal oxides employed in inorganic systems, offering an advantage when considering renewable/sustainable technologies.

Disulfides are one class of redox-active organic compounds that have been explored as possible alternatives to metal oxide cathode materials for lithium-ion batteries. The disulfide bond undergoes a reversible 2-electron reduction process (Figure 1a), making it an attractive candidate for energy-dense cathode materials.^3^ Furthermore, the electrochemical properties of disulfides can be tuned via the selection of neighboring electron-withdrawing or -donating groups. As such, a multitude of disulfide compounds have been investigated with an eye toward battery applications, including soluble dimers and oligomers, linear polymers, and networks.^4^ Through such studies, 2,5-dimercapto-1,3,4-thiadiazole (DMcT) and its analogues have garnered particular interest as organosulfur compounds for cathode active materials.^5−9^ Numerous studies over the years have demonstrated DMcT as an attractive candidate for lithium battery cathodes, switching between Li–S and disulfide states with high energy density and excellent cyclability. However, DMcT alone suffers from diffusion of the soluble active species away from the electrode, which degrades battery performance.^10−12^

**Figure 1:**
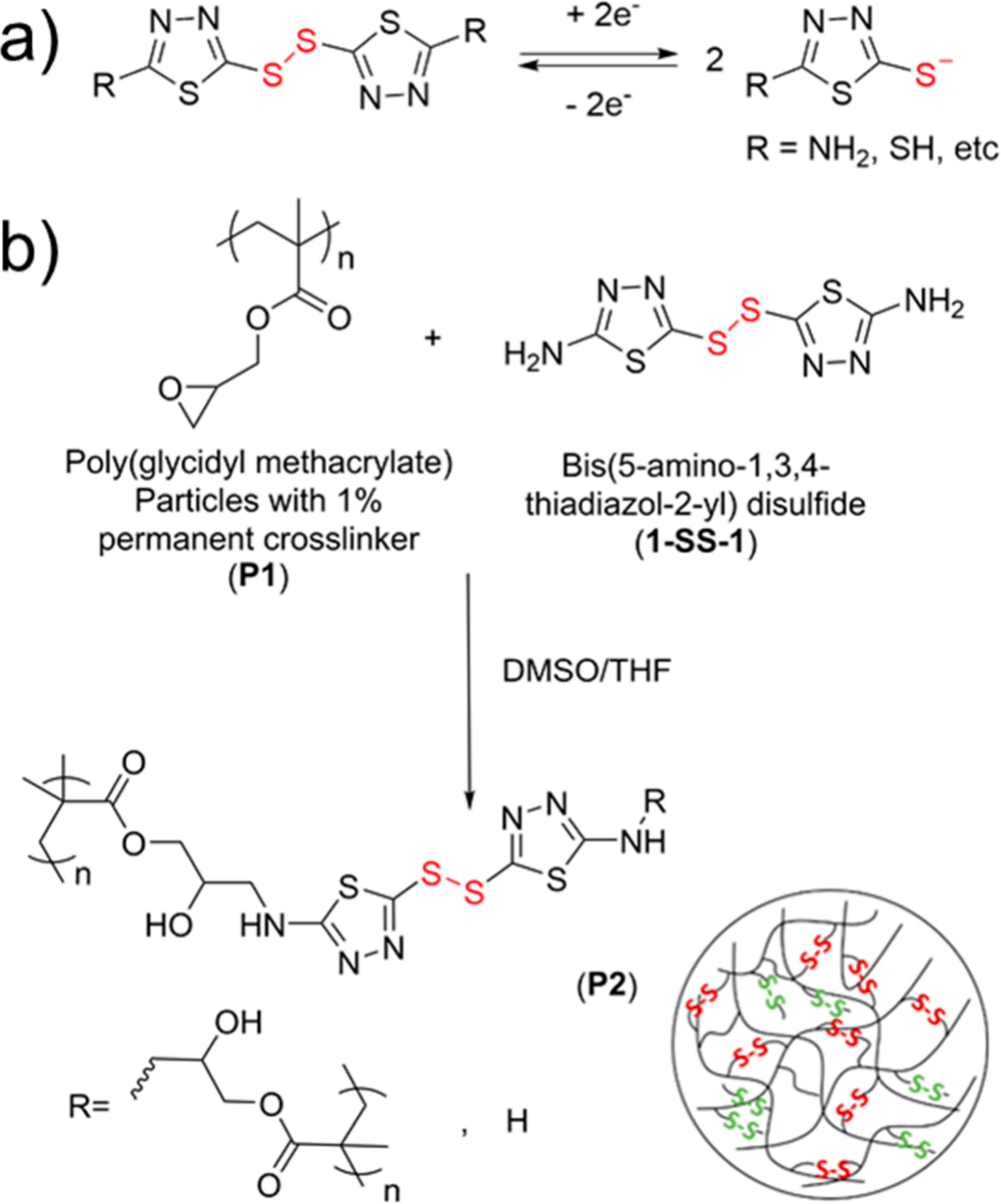
(a) Reversible electrochemical reaction of thiadiazole-based disulfides. (b) Reaction scheme for the synthesis of **1-SS-1**-functionalized PGMA particles (**P2**) from PGMA particles with a 1 wt % HMDA permanent cross-linker (**P1**).

Polymer-bound organosulfur compounds may offer a solution to this challenge. Covalently securing the organosulfur moiety to a scaffold material, such as a nonreactive polymer backbone, results in isolation of the electrode material to the cathode half cell. This approach has been investigated in films of DMcT-functionalized poly(3,4-ethylenedioxythiophene) (PEDOT).^9^ However, it was found that the conductivity of PEDOT proved insufficient to adequately charge the entire thickness of the film.^13^ A possible solution to this problem rests in the synthetic flexibility of polymer systems. The use of particulate morphologies for electrode-active materials results in dramatically increased surface area to volume ratios relative to films and other monolith morphologies and allows for simple blending with conductive additives, such as carbon black, to enhance electrical connectivity with the electrode substrate. This technique is utilized in current Li^+^-ion battery metal oxide electrodes and has been demonstrated with organic materials; for example, suspensions of polyaniline particles have been shown to charge more thoroughly than polyaniline films.^14^ While inorganic electrode materials must be milled to reduce their size, an energetically intensive process, colloidal polymerization techniques allow for facile synthesis of polymer particles with controlled size. Using particulate morphologies for polymeric redox-active materials has been successfully demonstrated in solid electrode batteries as well as in the emerging field of organic flow batteries.^15−22^ This particulate-based modality presents a potential route for simple, modular synthesis of effective electrodes with disulfide active chemistries.

Thus, the goal of this work is to explore the synthesis and redox behavior of disulfide-containing colloids with an eye toward their use in energy storage applications. Poly(glycidyl methacrylate) (PGMA) was selected as the matrix polymer for this study, as it is possible to access narrow dispersed colloidal particles using different polymerization techniques,^23^ and the epoxide group provides a functional handle for modular epoxy-amine “click” chemistry, allowing for a wide range of functional modifications to the polymer scaffold.^24^ Dispersion polymerization was used to produce particles with two targeted sizes (ca. 710 and 1670 nm in diameter, measured via scanning electron microscopy, SEM).^23^ Hexamethylene diamine (HMDA) (1 wt %) was reacted with these particles to produce cross-linked polymer microspheres (**P1**).^25^ Bis(5-amino-l,3,4-thiadiazol-2-yl) disulfide (**1-SS-1**) and bis(5-ethylamino-l,3,4-thiadiazol-2-yl) disulfide (**2-SS-2**) were selected as the disulfide-containing cross-linker and small-molecule analogue for this study and synthesized according to known procedures.^26^ Functionalization of **P1** with an excess of **1-SS-1** (5 equiv of **1-SS-1** per epoxy moiety) was carried out using a THF/DMSO cosolvent (tetrahydrofuran (THF) is a good solvent for PGMA, and dimethyl sulfoxide (DMSO) provides solubility for **1-SS-1**)^27^ to yield **P2** (Figure 1b). Successful synthesis of **P2** was determined by both Raman and FTIR. Raman of **P2** confirmed the presence of disulfides in the system (Figure S1a), while FTIR monitored the ring opening of the epoxides (Figure S1b).^28^ Based on this analysis, it was determined that roughly 88% of the available epoxide groups in **P1** reacted with **1-SS-1** to produce **P2** (Figure S2, Table S1).

Given the relatively low reactivity of the aromatic amine and the large excess of **1-SS-1** used in the reaction, it can be expected that a significant percentage of the reacted **1-SS-1** would only be monofunctionalized to the polymer (***x*-SS-1**) and thus not act as a cross-linker (Figure 1b, in green). In fact, dynamic light scattering of **P1** and **P2** showed that there is little to no change in the hydrodynamic diameter of the particles upon reaction with **1-SS-1**, consistent with this expectation (Table S3). Removal of these ***x*-SS-1** “loose ends” is critical to accessing electrochemically reversible redox particles, as cleavage of these groups would result in diffusion of the unbound redox-active molecules out of the particle.

It is known that disulfide bonds can undergo dynamic exchange with UV light.^29,30^ As such, it was hypothesized that removal of the ***x*-SS-1** could be achieved by irradiation of the particles to induce disulfide exchange and result in the formation of additional disulfide cross-links ***x*-SS-*x*** and free **1-SS-1**, which can diffuse into the solvent (Figure 2a). Exposing the particles to UV light (350 mW/cm^2^) in DMSO (which both swells the particles and is a good solvent for **1-SS-1**) results in the liberation of **1-SS-1** into the solution. To monitor the removal of **1-SS-1**, two UV annealing cycles were performed sequentially, and the supernatant was analyzed via UV–vis at 322 nm to determine the concentration of released **1-SS-1** (Figure S3). Following each UV irradiation cycle, the particle suspensions were centrifuged; the supernatant was poured off before being replaced by fresh DMSO; and the next irradiation cycle was performed. Results showed that no additional **1-SS-1** was detected in the solution during a second UV annealing process, consistent with the removal of ***x*-SS-1** from **P2** and the formation of the more densely cross-linked **P2-SS** containing only disulfide cross-linkers ***x*-SS-*x***. This is further supported by dynamic light scattering (DLS) of **P2-SS**, which showed a decrease in diameter to 1731 ± 109 nm in acetonitrile (ACN), which is ca. 21% smaller compared to **P2** (Figure 2b). In addition, the diameter of **P2-SS** was more consistent across different solvents (ACN, DMSO, and DMSO with salt) than **P1** or **P2**, consistent with a more dense cross-linking and demonstrating the effectiveness of the UV annealing technique (Table S3).

**Figure 2:**
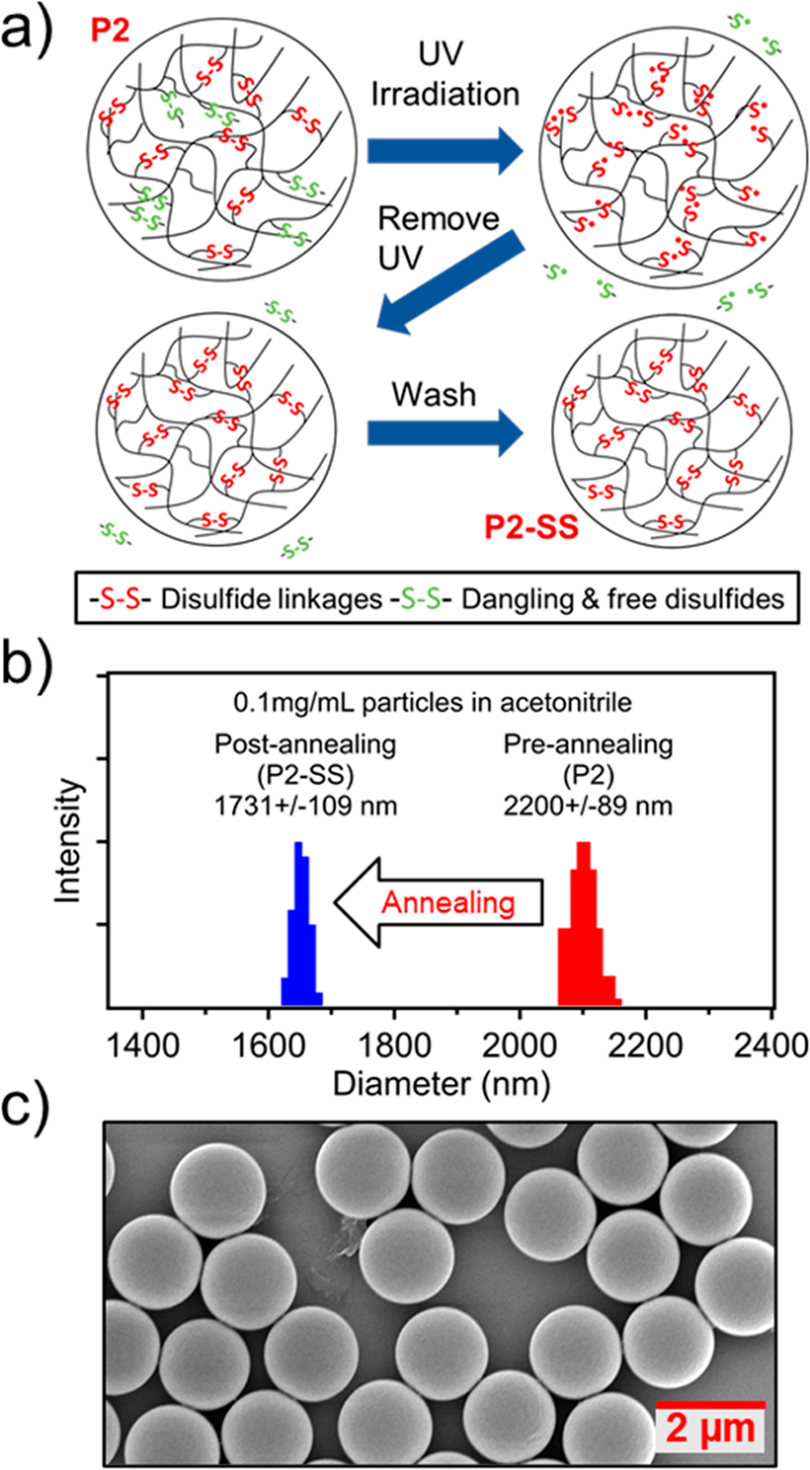
(a) Scheme for UV annealing of **P2** particles to remove dangling monofunctionalized ***x*-SS-1**. (b) Particle size reduction after UV annealing as measured by dynamic light scattering (DLS) in acetonitrile (ACN). (c) SEM image of **DS-RAP**.

Following functionalization with **1-SS-1**, approximately 12% of the PGMA epoxy groups remain unreacted within **P2-SS** based on FTIR integration data (Figure S2**)**. Epoxides are highly reactive electrophiles, and therefore it is desirable to passivate any residual groups. To this end, *N*-methylbutylamine was added to the particle and heated (Figure S4), where the removal of residual epoxides was confirmed by FTIR (Figure S1),^31^ resulting in stabilized disulfide-functionalized redox-active particles (**DS-RAP**) as shown in Figure 2c. DLS data showed that **DS-RAPs** swelled more in the organic solvents tested, presumably resulting from the addition of the aliphatic side groups (Table S3).

Cyclic voltammetry (CV) and galvanostatic cycling (GC) techniques were used to investigate the electrochemical properties of **DS-RAP** and compared to the soluble small-molecule analogue, bis(5-ethylamino-l,3,4-thiadiazol-2-yl) disulfide (**2-SS-2**). Carbon paper (CP) was selected as the working electrode as it allowed for the best electrochemical reversibility in CV experiments (Figure S5a). Specifically, solution CV of **2-SS-2** in ACN shows a reduction peak potential (*E*_p,r_) at −0.79 V vs Ag/Ag^+^ and an oxidation peak potential (*E*_p,o_) at −0.39 V vs Ag/Ag^+^. Samples for the CV of **DS-RAP** were obtained by drop-casting from an ethanol suspension onto CP electrodes resulting in 0.2 mg/cm^2^ of active material. **DS-RAP** exhibited an *E*_p,r_ at −0.69 V vs Ag/Ag^+^ and an *E*_p,o_ at −0.41 V vs Ag/Ag^+^ (Figure 3a), demonstrating a marked drop in peak to peak spacing (DE_p_) of 0.28 V relative to the DE_p_ of 0.40 V for **2-SS-2**. While the onset of the reduction process for both systems occurs at approximately −0.5 V vs Ag/Ag^+^, the more positive *E*_p,r_ position and sharper peak profile quantitively indicate faster electrochemical kinetics for the **DS-RAP** particles at the electrode surface. These values total to an approximate 30% overall reduction in DE_p_, suggesting the immobilization of ***x*-SS-*x*** on a polymer backbone as reversible cross-links lead to a considerable increase in electrochemical reversibility. This conclusion is supported by the far greater difference between reduction peak current density (*i*_p,r_) and oxidation peak current density (*i*_p,o_) in the in **2-SS-2** small molecule when compared to **DS-RAP**. This can be rationalized by the fact that the reductively cleaved small molecules are free to diffuse away from the electrode surface, preventing oxidation back to the disulfide during the return sweep (Figure 3a, inset i). In contrast, the disulfide moieties bound to the polymer are held in a relatively fixed position, keeping them in closer proximity to its cleaved partner (or other thiolates) and the electrode surface (Figure 3a, inset ii).

**Figure 3:**
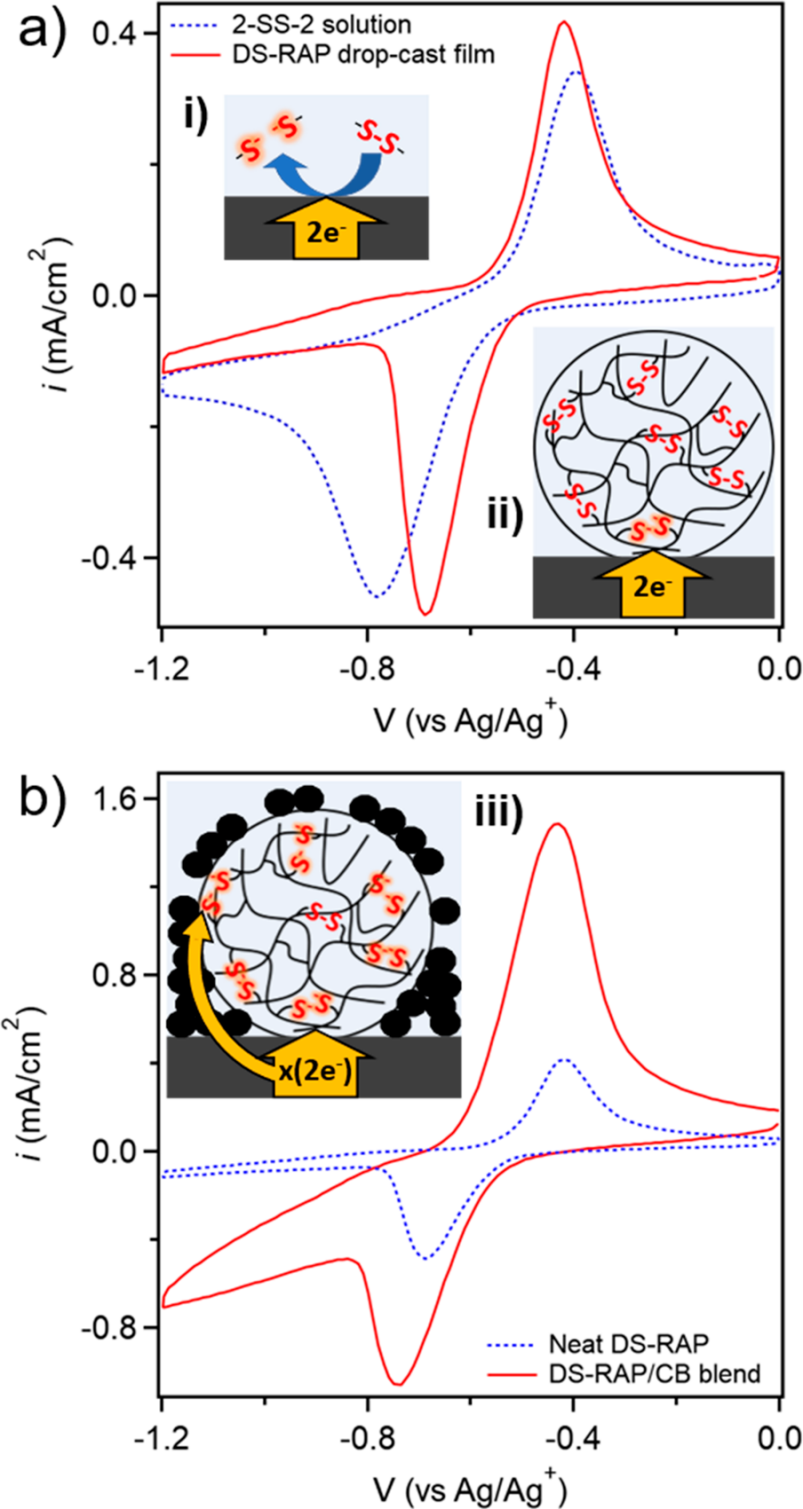
CV data with inset schemes. 100 mM tetrabutylammonium hexafluorophosphate (TBAPF_6_)/ACN electrolyte, with 20 mV/s scan rate. Potentials are relative to Ag/Ag^+^ nonaqueous reference electrodes. (a) **2-SS-2** solution and drop-cast **DS-RAP**. (b) Enhanced electrochemical response of **DS-RAP** by 1:1 by weight blending of Super P carbon black (CB).

A second reductive process is observed in the particle system starting at approximately −0.8 V vs Ag/Ag^+^ and extending out to the maximum scan value of −1.2 V vs Ag/Ag^+^. To eliminate the possibility that the second reductive process is the result of irreversible reductive degradation, control CV experiments were performed on a series of differently functionalized particles. **P1** particles lacking ***x*-SS-*x*** showed no appreciable signal along the reduction sweep (Figure S5b). Additionally, a batch of **P1** was reacted with excess HMDA cross-linker to ring open the epoxide, resulting in densely cross-linked particles with redox-inert cross-linkers. These particles showed no electrochemical reactivity in the potential window of interest. As such, these control experiments suggest that both reductive processes in the **DS-RAP** particles are a consequence of the reductive cleavage of ***x*-SS-*x***.

The **DS-RAP** particles investigated in this study are electronically insulating. Thus, to increase electronic access, the **DS-RAPs** were blended with Super P carbon black (CB) particles (1:1 by weight). For these blended samples, *i*_p,r_ increased by an approximate factor of 2, while the reduction current density increased more than five times across the potential range of −0.8 to −1.2 V vs Ag/Ag^+^. The value of *i*_p,o_ was shown to increase much more than *i*_p,r_, providing evidence that the reductive process at lower potentials is the result of continued disulfide reduction into the depth of the particle (Figure 3b). It is possible that the dynamic nature of the disulfide bond means the reductive process is not self-limiting at the surface. For example, thiolate–disulfide exchange reactions could result in disulfide bonds effectively moving from the core of the particle to the surface, thus promoting additional electrochemical reactions. Overall, these CV results show that the DS-RAP particles blended with CB particles in the electrode are more readily accessed electrochemically.

Galvanostatic cycling (GC) experiments were performed to test the feasibility of **DS-RAPs** as an energy storage material. Electrodes were prepared by casting 0.4 mg of 1:1 **DS-RAP**:CB from ethanol onto 1 cm^2^ of CP. All GC experiments were performed at a C-rate of 0.25 C (3.95 μA/cm^2^) with potential limits of −1.2 to 0 V vs Ag/Ag^+^ (ca. 2.4 to 3.6 V vs Li/Li^+^) using a 3-electrode electrochemical cell. Figure 4a shows a representative charge/discharge profile, while Figure 4b shows capacity and efficiency over multiple cycles. The **DS-RAP** synthesized in this study contains an estimated 39% ***x*-SS-*x*** by mass (compared to a theoretical maximum of 48%, assuming two epoxides per ***x*-SS-*x***) (Figure S6), leading to a theoretical specific capacity of 79 mAh/g_particle_, assuming a two-electron reduction process (eqs S1 and S2). **DS-RAPs** were initially tested using 1 M TBAPF_6_ in ACN as the electrolyte. The initial cycle yielded a specific discharge capacity (SDC) of 4.81 mAh/g and Coulombic efficiency (CE) of 88.4%. However, this system demonstrated a sharp initial decrease in capacity, along with a further decrease over progressive cycles, resulting in an SDC of 2.97 mAh/g_particle_ and CE of 83.7% at cycle 50 (Figure S7b).

**Figure 4:**
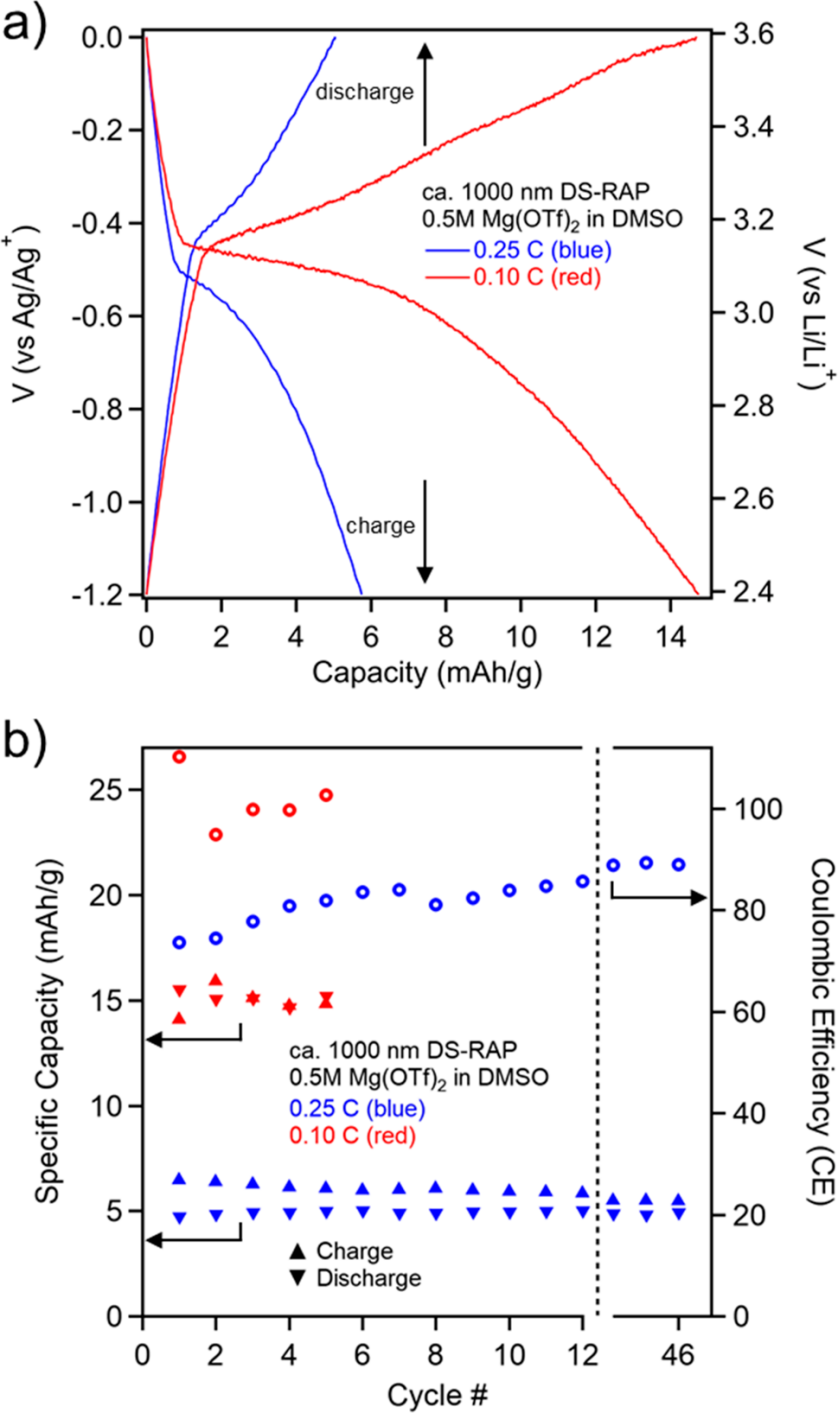
Galvanostatic cycling (GC) of the smaller **DS-RAP** (ca. 1000 nm) at a C-rate of 0.10 and 0.25 C. (a) Charge/discharge curves (cycle 3 for 0.10 C and cycle 46 for 0.25 C). Potential (V) vs Li/Li^+^ is converted from Ag/Ag^+^. (b) Charge/discharge cycling stability and Coulombic efficiency (CE) data. The reported GC experiments were performed using DMSO with 0.5 M Mg(OTf)_2_. Additional GC results are shown in the Supporting Information (Figure S6).

Alternative solvents were explored for the electrolyte as a strategy to improve cycling stability. Of the multiple solvents investigated (Table S4), DMSO yielded stable cycling across the full 50 cycle range. The initial cycle yielded an SDC of 1.65 mAh/g and CE of 86.1%. At cycle 50, the SDC remained stable at 1.65 mAh/g along with an improvement in CE to 92.7% (Figure S7b). Comparison of the GC results between ACN and DMSO indicates that ACN is likely promoting an irreversible electrochemical reaction that leads to the capacity fade, while contributing to a portion of the specific charging capacity. While the cycling stability is improved in DMSO, the resulting SDC remains below that obtained in ACN. In order to address the low specific capacity of DS-RAP, the effect of particle size and supporting electrolyte salt selection was examined next.

As a route to further increase the specific capacity, **DS-RAPs** with a smaller diameter of ca. 711 nm (dry diameter via SEM) were synthesized (Figure S8) and cycled using TBAPF_6_/DMSO electrolyte. This particle size approaches the lower limit at which **DS-RAP** can be synthesized using the employed dispersion polymerization method. The new **DS-RAP** swelled to 1034 ± 57 nm in diameter in TBAPF_6_/DMSO, compared to 1867 ± 46 nm for the larger particle. Notably, the GC results indicate that the SDC and CE are 3.32 mAh/g and 89.5% at cycle 50, respectively. The 44.6% smaller **DS-RAP** exhibited double the SDC of the larger **DS-RAP** in the presence of TBAPF_6_/DMSO while maintaining similar CE at cycle 50 (Figure S7d). Moreover, the smaller **DS-RAP** maintains stable cycling across the full 50 cycles, similar to the larger **DS-RAP** along with the particle integrity maintained before and after cycling (Figure S9). The smaller particles reduce the characteristic diffusion length and have larger surface area to volume ratios, resulting in a larger portion of the particle being electrochemically accessible as indicated by the improved SDC.

The selection of salt cation for the electrolyte was also shown to impact the cycling capacity. While TBAPF_6_ is stable in electrochemical applications and highly soluble in many organic solvents, the bulky organic cation can limit diffusion into the particle. LiPF_6_ and KPF_6_ were investigated but found to exhibit reduced electrochemical reversibility due to the greater positive potential needed to reoxidize the thiolates (Figure S5c). Batteries based on divalent cations, such as Mg^2+^, continue to garner interest on account of their higher theoretical energy densities. Interestingly, unlike LiPF_6_, magnesium triflate (Mg(OTf)_2_) did not show substantially increased peak spacing relative to TBAPF_6_ (Figure S5d). Thus, Mg(OTf)_2_ was selected as an alternative to TBAPF_6_. A 0.5 M Mg(OTf)_2_ DMSO solution was used on account of its practical solubility limits. By using the Mg(OTf)_2_/DMSO electrolyte, the GC experiments showed a SDC and CE of 4.94 mAh/g and 89.0% at cycle 46, respectively (Figure S7f, Table S5). This SDC is a 49.7% increase compared to the SDC when using TBAPF_6_/DMSO, which could be explained, at least in part, by the divalent Mg^2+^ cation being able to neutralize two thiolate anions, reducing the amount of cations needed to diffuse into the polymer matrix.

Given the relatively slow charge-transfer kinetics of the disulfide redox couple,^5^ the C-rate was lowered from 0.25 C used in the screening experiments to 0.10 C, resulting in an SDC of 15.21 mAh/g after 5 cycles, increasing the capacity by over three times to 19.25% of the theoretical. This result is in line with efficiencies from conceptually similar materials from recent works.^9,32^

In summary, this work demonstrates a simple synthetic approach for disulfide-containing redox-active particles. Through epoxy-amine click chemistry, PGMA particles, prepared using dispersion polymerization, were functionalized with bis(5-amino-1,3,4-thiadiazole-2-yl) disulfide, in which the disulfide behaves as a reversible cross-linker, resulting in densely cross-linked particles with a theoretical capacity of 79 mAh/g. Cyclic voltammetry revealed that the incorporation of the disulfide into the particle resulted in improved electrochemical reversibility of the redox couple compared it its small-molecule equivalent, reducing CV peak spacing by 30%. Moreover, galvanostatic cycling demonstrated potential use of this material as an energy storage material, using a Mg-based electrolyte to achieve high cycling stability, obtaining 15.21 mAh/g at 0.1 C, with high Coulombic efficiency. A specific discharge capacity reaching 19.25% that of the theoretical appears to be limited largely by particle size, offering a direction for future improvement. Overall, this work shows a successful demonstration of a modular RAC platform and provides a tool for the design of organic battery materials, where spatial confinement of the redox-active material is a key consideration.

## Supporting Information Available

Experimental procedures, FT-IR, Raman spectroscopy, and FT-IR fitting data for particle synthesis, supporting electrolyte viscosities, particle DLS, UV annealing data, reaction scheme for epoxy ring opening, CV data for substrate and electrolyte selection, particle UV–vis, GCPL data with a summary of results, small particle SEM, and qualitative observations (PDF)
