# Supplementary material for: Synthesis and Characterization of Redox-Responsive Disulfide Cross-Linked Polymer Particles for Energy Storage Applications
Source: ACS Macro Lett. 2021 Dec 9;10(12):1637–42. doi: 10.1021/acsmacrolett.1c00682 (PMC8697551; doi:10.1021/acsmacrolett.1c00682)
Supplement: Supplementary file 1 — mz1c00682_si_001.pdf [file mz1c00682_si_001.pdf]

Supporting Information

Synthesis and characterization of redox-responsive disulfide-crosslinked polymer particles for energy storage applications

Garrett L. Grocke,[1,2] Hongyi Zhang,[1,2] Samuel S. Kopfinger,[1] Shrayesh N. Patel,[1,2,3*] Stuart J. Rowan[1,2,3,4*]

1. Pritzker School of Molecular Engineering, University of Chicago, Chicago, Illinois 60637, USA
2. Joint Center for Energy Storage Research, Argonne National Laboratory, Argonne, IL 60439, USA
3. Chemical Sciences and Engineering Division, Argonne National Laboratory, Argonne, IL 60439, USA
4. Department of Chemistry, University of Chicago, Chicago, Illinois 60637, USA

*Corresponding authors email –shrayesh@uchicago.edu; stuartrowan@uchicago.edu

Contents:

Experimental procedures	S2
**Figure S1**. Fourier-transform infrared spectroscopy (FT-IR) and Raman spectroscopy of bis(5-N-amino-l,3,4-thiadiazol-2-yl) disulfide (**1-SS-1**), unfunctionalized PGMA particles (**P1**), **P1** functionalized with **1-SS-1 (P2)** and followed by UV annealing (**P2-SS**), and **P2-SS** reacted with N-methylbutylamine (**DS-RAP**)	S6
**Figure S2**. FT-IR curve fitting of the carbonyl and epoxy peaks in **P1** and **P2-SS**	S7
**Table S1**. Peak area integration data for **P1** and **P2-SS** FT-IR Voight curve fitting.	S8
**Table S2.** Viscosity values of electrolyte solvents for DLS measurement parameters	S9
**Table S3.** Particle size at different synthetic stages determined by dynamic light scattering (DLS) measurements in selected solvents and scanning electron microscopy (SEM) of dry particles.	S10
**Figure S3**. Quantification of **1-SS-1** removed from **P2** particles during UV annealing to produce **P2-SS**	S11
**Figure S4.** Reaction scheme for epoxy ring-opening in **P2-SS** particles using *N*-methylbutylamine to produce **DS-RAP**.	S12
**Figure S5**. Electrochemical characterization data for electrode selection, electrochemical reactivity of system components, and cycling with different electrolytes	S13
**Figure S6**. Determination of particle-functionalized bis(5-N-amino-l,3,4-thiadiazol-2-yl) content **x-SS-x** by UV-Vis absorption	S15

**Figure S7**. Galvanostatic cycling with potential limits (GCPL) data for **DS-RAP** using different electrolyte solvents and **DS-RAP** particle sizes	S16
**Table S4**. Qualitative observations for screening of non-selected electrolyte solvents.	S17
**Figure S8**. Reduced size particle DLS size change characterization and SEM imaging	S18
**Table S5**. Summary **DS-RAP** specific discharge capacity (SDC) and Coulombic efficiency (CE) results under different conditions (0.25C C-rate)	S19
**Figure S9**. SEM imaging of electrodes containing **DS-RAP** blended with carbon black	S20
**References**	S21

Experimental Procedures.

Materials: Glycidyl methacrylate (GMA) containing inhibitor, 5-amino-1,3,4-thiadiazole-2-thiol, hydrogen peroxide solution (30% w/w) in aqueous solution containing inhibitor; polyvinylpyrrolidone (PVP) (average mol. wt 40,000), hexamethylenediamine, 4-ethyl-3-thiosemicarbazide, and carbon disulfide were purchased from Sigma-Aldrich. All other reagents were purchased from Fisher Scientific. All reagents were used without further purification unless mentioned.

Synthesis of PGMA particles (P1): Monodisperse PGMA particles (**P1**) were synthesized via dispersion polymerization. Two sizes were prepared using the following procedures:

Larger particles: Inhibitor was removed from GMA monomer by passing through a basic alumina column. 4 g PVP was dissolved in 102.7 mL of absolute ethanol and 7.42 mL of deionized water in a 250 mL round-bottom flask. The solution was heated to 70°C and stirred at 165 rpm. The reaction vessel was sealed and purged for 20 min using filtered dry Ar gas. 0.2 g of AIBN was dissolved in 9.5 mL of GMA monomer and charged to the reaction vessel. The vessel was kept purged for an additional 10 minutes to ensure adequate removal of oxygen. The solution was reacted for 12 hours. The resulting particles were centrifuged, decanted, and washed with methanol. The process was repeated 3 times. The particles were then dried under vacuum and 9.04 g of product was obtained (89% yield).

Smaller particles were synthesized using similar procedures, with the following changes: The solvent was replaced by pure ethanol (100 mL), the monomer feed amount was decreased by half (4.75 mL) and the stirring speed was increased (320 rpm). 4.7 g of particles was obtained (92% yield).

Addition of permanent crosslinkers to PGMA particles: 4 g of particles was redispersed in 50 mL of EtOH in a bath sonicator. 1 wt% (40 mg) of hexamethylene diamine (HMDA) (relative to particle mass) was added to the dispersion in the form of 10 mg/mL HMDA ethanol solution and reacted at 50°C for 24 hours. Particle size was measured by DLS and SEM. The resulting particles

were dispersed in EtOH and freeze-dried before further functionalization. Same procedure was used on smaller particles.

Synthesis of disulfides (1-SS-1 and 2-SS-2): 12 g of 5-amino-l,3,4-thiadiazol-2-thiol was dissolved in 1000 mL of methanol while stirring. 90 mL of 30% aqueous hydrogen peroxide was added dropwise over 45 min. A resulting yellow solid was formed after approximately 2 hours, which was collected by filtration and washed extensively with methanol before being dried under vacuum overnight to yield ca. 11g of bis(5-amino-l,3,4-thiadiazol-2-yl) disulfide (**1-SS-1**) was obtained with 91.6% yield. ^{1}H NMR (400 MHz, DMSO-d6) δ 7.76 (s, 4H). ^{13}C NMR (400 MHz, DMSO-d6) δ 172.68, 149.15.

11.9 g (0.1 mol) of 4-ethyl-3-thiosemicarbazide was added into 130 mL of EtOH with 4g (0.1 mol) of sodium hydroxide. 7.6 g (0.1 mol) of carbon disulfide was added to the solution and heated to reflux overnight. The solution was dried in vacuum and yellow solid was obtained. The yellow solid was dissolved in 30 mL of water. Concentrated HCl was added dropwise until the pH of solution was below 2. A white precipitated was formed and filtered. The precipitate was washed with water and dried under vacuum. 11.5 g of product was obtained (71% yield). ^{1}H NMR (400 MHz, DMSO-d6) δ 13.27 (s, 1H), 7.54-7.51 (t, 1H), 3.18-3.12 (m, 2H), 1.14-1.10 (t, 3H). ^{13}C NMR (400 MHz, DMSO-d6) δ 180.89, 161.20, 38.78, 14.45.

Bis(5-ethylamino-l,3,4-thiadiazol-2-yl) disulfide (**2-SS-2**) was synthesized from the above product by the same oxidation procedure as 1-SS-1 with 65% yield. ^{1}H NMR (400 MHz, DMSO-d6) δ 8.26-8.23 (t, 2H), 3.36-3.29 (m, 4H), 1.20-1.16 (t, 6H). ^{13}C NMR (400 MHz, DMSO-d6) δ 172.56, 149.02, 40.05, 14.58.

Functionalization of P1 with 1-SS-1 to produce P2: 1 g (7 mmol) of **P1** particles were dispersed in 50 mL of THF via bath sonication. **1-SS-1** (9.30 g, 35 mmol) was dissolved in 100 mL of DMSO and charged into a reaction vessel. The reaction was carried out at 66°C at 300 rpm for 6 days. The particles were then washed with fresh 1:1 DMSO/methanol solution and centrifuged at 4000 rpm for 15 min, after which the supernatant decanted and the washing process repeated 6 times until no unreacted **1-SS-1** was released into the decanted supernatant, as measured by UV-Vis at 325 nm, attributed to the **1-SS-1** small molecule in solution.

UV annealing to remove monofunctionalized 1-SS-1 from P2 to produce P2-SS: A calibration curve was constructed by serial dilution of **1-SS-1** solution in DMSO measured via UV-Vis at 325 nm. 500 mg of **P2** was added to 50mL of DMSO to prepare a 10 mg/mL particle dispersion. The **P2** dispersion was sonicated with a bath sonicator for greater than 1 hour. The vial was irradiated with a UV source at 350 mw/cm^2 while stirring. 1 mL of dispersion was taken every 20 min and centrifuged at 6000 rpm to pellet particles. 40 μL of decanted supernatant was taken and was passed through a 0.45 μm filter while the particle pellet was redispersed by bath sonicator and recombined into the bulk dispersion with the addition of 40 μL fresh DMSO. The filtered decanted solvent was then diluted 10 times and measured by UV-Vis. By monitoring the peak at 325 nm, the amount of **1-SS-1** small molecule released to the supernatant from UV annealing process could

be determined based on the calibration curve. The process was continued until the amount of **1-SS-1** in solution plateaued. After the first annealing process, the particles were washed and centrifuged again with 1:1 methanol and DMSO. The signature peak of **1-SS-1** was not detectable during a second UV annealing process. After washing and centrifugation with methanol and water, the particles (**P2-SS**) were dried under vacuum before secondary amine functionalization.

N-methylbutylamine functionalization of P2-SS to produce DS-RAP: 500 mg of **P2-SS** was dispersed in 50mL of EtOH in a bath sonicator for 40 min until a homogeneous dispersion was obtained. 500 mg (5.7 mmol, 9 eq. relative to the 12% remaining **P2-SS** residue epoxides calculated by FTIR integration) of N-methylbutylamine was added to the reaction vessel and the reaction was carried at 50°C for 2 days. The product was centrifuged, washed with methanol, and the supernatant was decanted. Resulting **DS-RAP** particles were dried under vacuum overnight. The removal of remaining epoxides was confirmed by FT-IR and the preservation of disulfide was confirmed by Raman.

Synthesis of P1-HMDA control particles: 50 mg (~0.35 mmol GMA repeating units) of dry larger PGMA particles without permanent crosslinker were dispersed in 10 mL of ethanol and sonicated for 1 hour. 20 mg (0.175 mmol) of HMDA was added in the form of 10 mg/mL HMDA ethanol solution. The reaction was carried out at 300 rpm and 70 ^{o}C for 1 day. The resulting particle dispersion was centrifuged, decanted, and washed with methanol. The process was repeated 3 times. The particles were then dried under vacuum and 40.6 mg of product was obtained (57% yield).

Dynamic Light Scattering: DLS measurements were performed using a Brookhaven Instruments BI-200SM. The dry particle samples were dispersed in a 1 mg/mL concentration using bath sonication for a minimum of 1 hour and then diluted to 0.1 mg/mL dispersion prior to measurement. The viscosity parameters of electrolyte solution used for DLS was measured by a TA Instruments ARES-G2 shear rheometer using a cup geometry with a shear rate range from 1 to 200 s^{-1}. Applicable viscosity data can be found in **Table S2**.

UV-Vis Spectroscopy: A Shimadzu UV-3600 Plus UV-Vis-NIR spectrophotometer was used to measure from 250 nm to 500 nm with a sampling interval of 1 nm. All sample solutions or dispersions were made using DMSO as the supporting solvent. 0.05 mg/mL suspensions of DS-RAP were used for particle characterization.

FT-IR: Shimadzu IRTracer-100 FT-IR with ATR diamond was used to characterize **P1**, **P2-SS**, and **DS-RAP**. A minimum of 10 mg particle powder was placed directly onto the attenuated total reflection (ATR) crystal. The amount of unreacted epoxide group was quantified by integrating peaks 846 cm^{-1} and 905 cm^{-1}, corresponding to symmetric and asymmetric stretching of the epoxide group. The signal was normalized to the amount of PGMA monomer by integrating the peak at 1760 cm^{-1} corresponding to the PGMA carbonyl. Peaks were fit to a Voight model using Igor Pro multi-peak fitting.

Raman: A Horiba LabRAM HR Evolution confocal Raman microscope was used with 50X LWD objectives, 10% filter, and 532 nm laser for Raman measurements. Sample powders (ca. 10 mg per sample) were pressed into a pellet on standard glass microscope slide.

Scanning Electron Microscopy:

Bare particles: Silicon substrate was cut into 1cm×1cm squares and washed via sonication in acetone followed by methanol. 50 microliters of 1 mg/mL particle dispersions in ethanol were cast onto the silicon substrates and allowed to air dry.

Composite electrodes: 1:1 **DS-RAP**/CB samples containing a total of 0.2 mg **DS-RAP** were cast onto carbon paper electrodes. After galvanostatic cycling, electrodes were cleaned by soaking in clean acetonitrile (ACN) twice followed by drying in a convection oven at 60°C.

A Cressington 208HR Sputter Coater was used to coat 8 nm of Pt/Pd to prevent sample surface charging during imaging. A Carl Zeiss Merlin SEM was used to image the particles samples at 3 kV EHT.

Cyclic Voltammetry (CV): A Biologic SP-200 potentiostat was used for all electrochemical measurements. All tests were performed using a standard 3-electrode electrochemical cell. 1cm^2 of Sigracet GDL 39 AA carbon paper (CP) was selected as the working electrode from a selection of different porous carbon substrates based on the relative reversibility of the **1-SS-1** redox couple (**Figure S5a**). The CP was attached to a Pt wire electrode (CH Instruments CHI115) by threading the wire through two small holes created near opposing edges of the CP sample. The counter electrode was a bare Pt wire. A non-aqueous Ag/Ag^+ reference electrode was used, comprised of a silver wire immersed in 0.01 M $AgNO_3$ and 0.1 M tetrabutylammonium hexafluorophosphate ($TBAPF_6$) in ACN. 20 μL of **DS-RAP** particles in a 10 mg/mL ethanol suspension (0.2 mg total **DS-RAP** mass) were drop-cast onto CP electrodes and allowed to dry. For blended samples, Super P carbon black (CB) was mixed with the **DS-RAP** in ethanol in a 1:1 mass ratio. 40 μL of the 1:1 suspension, containing 5 mg/mL of each **DS-RAP** and CB, were drop-cast onto CP electrodes, resulting in the same 0.2 mg total **DS-RAP** mass. All data presented was measured at 20 mV/s. Solvents for electrochemical experiments were degassed by sparging with Ar gas. All salts were used as-received and stored under Ar gas.

Galvanostatic Cycling (GC) Experiments: Samples were prepared in a similar manner to CV experiments. 1:1 **DS-RAP**/CB samples containing a total of 0.2 mg **DS-RAP** were cast onto CP electrodes and immersed in selected supporting electrolyte. A bare carbon felt electrode was attached to a Pt wire for use as the counter electrode, and a nonaqueous Ag/Ag^+ reference electrode was used. Samples were equilibrated for 12 hours prior to cycling.

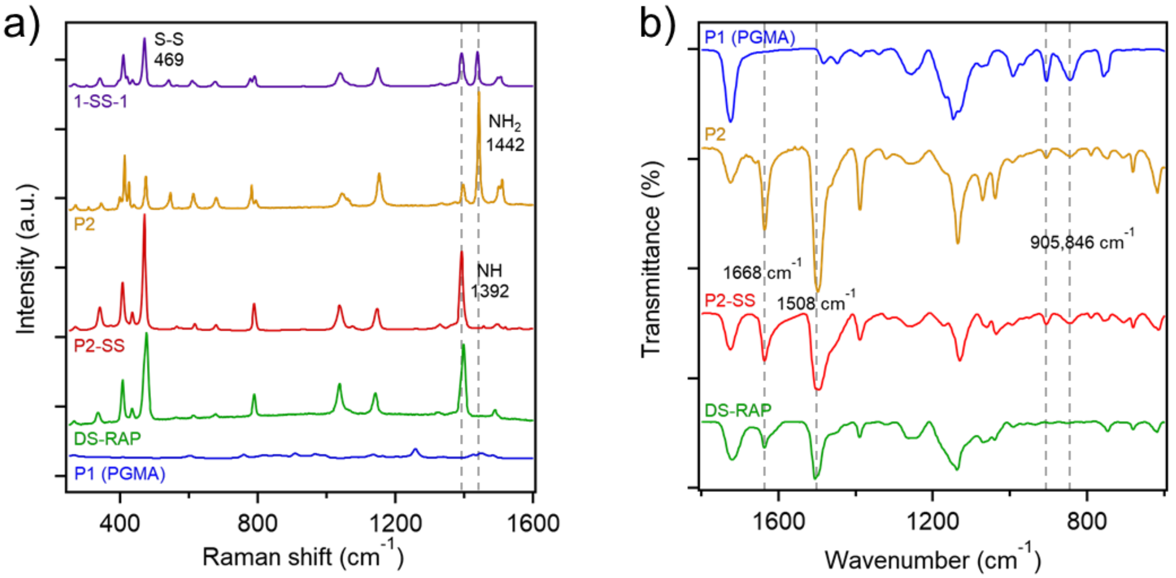

Figure S1. Raman and FT-IR spectra of **P1**, **1-SS-1**, **P2-SS**, and **DS-RAP**. **a)** Raman spectra and **b)** FT-IR spectra with labeled peaks of interest.

Raman of **DS-RAP** confirms the presence of disulfide bonds via an observed characteristic peak at 469 cm^{-1}, indicating their successful retention in the particle system after UV annealing, and providing evidence for the covalent attachment of **1-SS-1** to the PGMA polymer backbone. The lack of a prominent characteristic NH_2 amine peak at 1442 cm^{-1} and concurrent increase of NH amine peak at 1392 cm^{-1} in **DS-RAP** provide further confirmation of functionalization of the disulfide moiety onto particles and removal of monofunctionalized **1-SS-1** from **P2**.

FTIR peaks centered at 846 cm^{-1} and 905 cm^{-1} correspond to the symmetric and asymmetric stretching of the epoxide group, respectively. A characteristic carbonyl peak at 1722 cm^{-1} was observed for particles of all functionalization state. Peaks centered at 1668 cm^{-1} and 1508 cm^{-1} are attributed to N-H bending resulting from amination of epoxide groups.

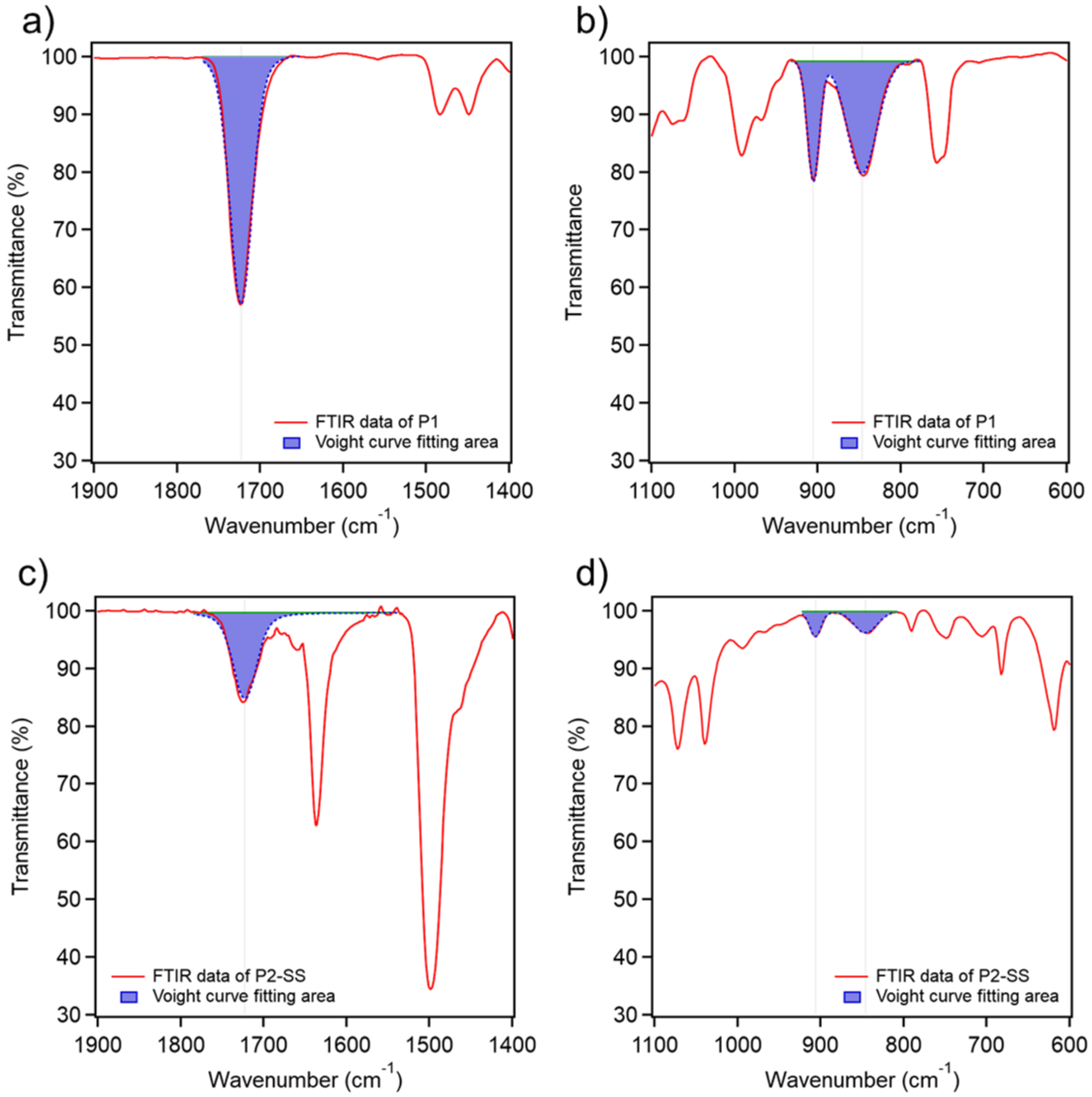

Figure S2. FT-IR Voight curve model fitting of **a)** carbonyl group of **P1**, **b)** epoxide group of **P1,** **c)** carbonyl group of **P2-SS,** and **d)** epoxide group of **P2-SS**.

Table S1. Peak area integration data for **P1** and **P2-SS** FT-IR Voight curve model fitting.

	**Area (1722 cm^{-1}) (carbonyl)**	**Normalization factor (α)**	**Area (846 cm^{-1}) (symm. epoxide)**	**j = α*area (846 cm^{-1})**	**% res. epoxide (j_X/j_{PI})**
P1	972	1	674	674 (j_{P1})	1
P2-SS	1168	1.202	108	89.877 ($j_{PS\text{-}SS}$)	0.133
	〃	〃	**Area (906 cm^{-1}) (asymm. epoxide)**	**α*area(906 cm^{-1})**	**% res. epoxide (j_X/j_{PI})**
P1	〃	〃	267.89	267.89 (j_{P1})	1
P2-SS	〃	〃	36	29.959 ($j_{PS\text{-}SS}$)	0.112
**% residual epoxide (estimated from average) = 12.25%**					

Peak area values for carbonyl stretching (1722 cm^{-1}), epoxide symmetric bending (846 cm^{-1}), and epoxide asymmetric bending (906 cm^{-1}) were obtained from FT-IR profiles. Data was normalized to the carbonyl peak area for **P1**. The amount of residual epoxide in **P2-SS** after **1-SS-1** functionalization was determined by the average change in normalized epoxide peak area for the symmetric and asymmetric bending peaks.

The area of the carbonyl stretching peak was used as the basis of normalization for amount of particle present in each measurement, as the amount of carbonyl is directly correlated to the amount of PGMA in sample. The area of each epoxide peak was normalized by this factor, yielding value j. The percent remaining epoxide was determined by dividing the normalized epoxide peak area after amination by the normalized value for the pre-amination sample.

Table S2. Viscosity values of electrolyte solvents for DLS measurement parameters

**Solvent**	**Viscosity**	**Reference**
ACN	0.334 cP (mPa*s)	1
ACN, 1M $TBAPF_6$	0.799 cP (mPa*s)	Measured for this work
DMSO	1.991 cP (mPa*s)	2
DMSO, 1M $TBAPF_6$	4.490 cP (mPa*s)	Measured for this work
DMSO, 0.5M $Mg(OTf)_2$	5.393 cP (mPa*s)	Measured for this work

Table S3. Particle diameters from DLS measurements for **P1**, **P2**, **P2-SS**, and **DS-RAP** in different solvents and electrolytes.

**Larger particle state**	**P1 (nm)**	**P2 (nm)**	**P2-SS (nm)**	**DS-RAP (nm)**
Dry (via SEM)	1672 ± 33	1685 ± 47	1680 ± 21	1723 ± 65
ACN	2198 ± 142	2200 ± 89	1731 ± 109	1894 ± 85
ACN w/ 1M $TBAPF_6$	2086 ± 142	2058 ± 99	1738 ± 69	1956 ± 45
DMSO	2149 ± 138	2364 ± 54	1793 ± 105	1903 ± 130
DMSO w/ 1M $TBAPF_6$	2035 ± 100	2018 ± 117	1736 ± 130	1867 ± 46
DMSO w/ 0.5M $Mg(OTf)_2$	2162 ± 130	2160 ± 229	1734 ± 83	1885 ± 27
**Smaller particle state**	**P1 (nm)**	**P2 (nm)**	**P2-SS (nm)**	**DS-RAP (nm)**
Dry (via SEM)	711 ± 10	705 ± 20	694 ± 2	796 ± 13
ACN	1316 ± 48	1298 ± 89	716 ± 168	1338 ± 26
ACN w/ 1M $TBAPF_6$	1241 ± 32	1381 ± 86	776 ± 69	1270 ± 113
DMSO	1408 ± 88	1377 ± 63	776 ± 78	1011 ± 73
DMSO w/ 1M $TBAPF_6$	1348 ± 138	1402 ± 56	738 ± 74	1034 ± 57
DMSO w/ 0.5M $Mg(OTf)_2$	1371 ± 60	1416 ± 77	783 ± 61	1013 ± 44

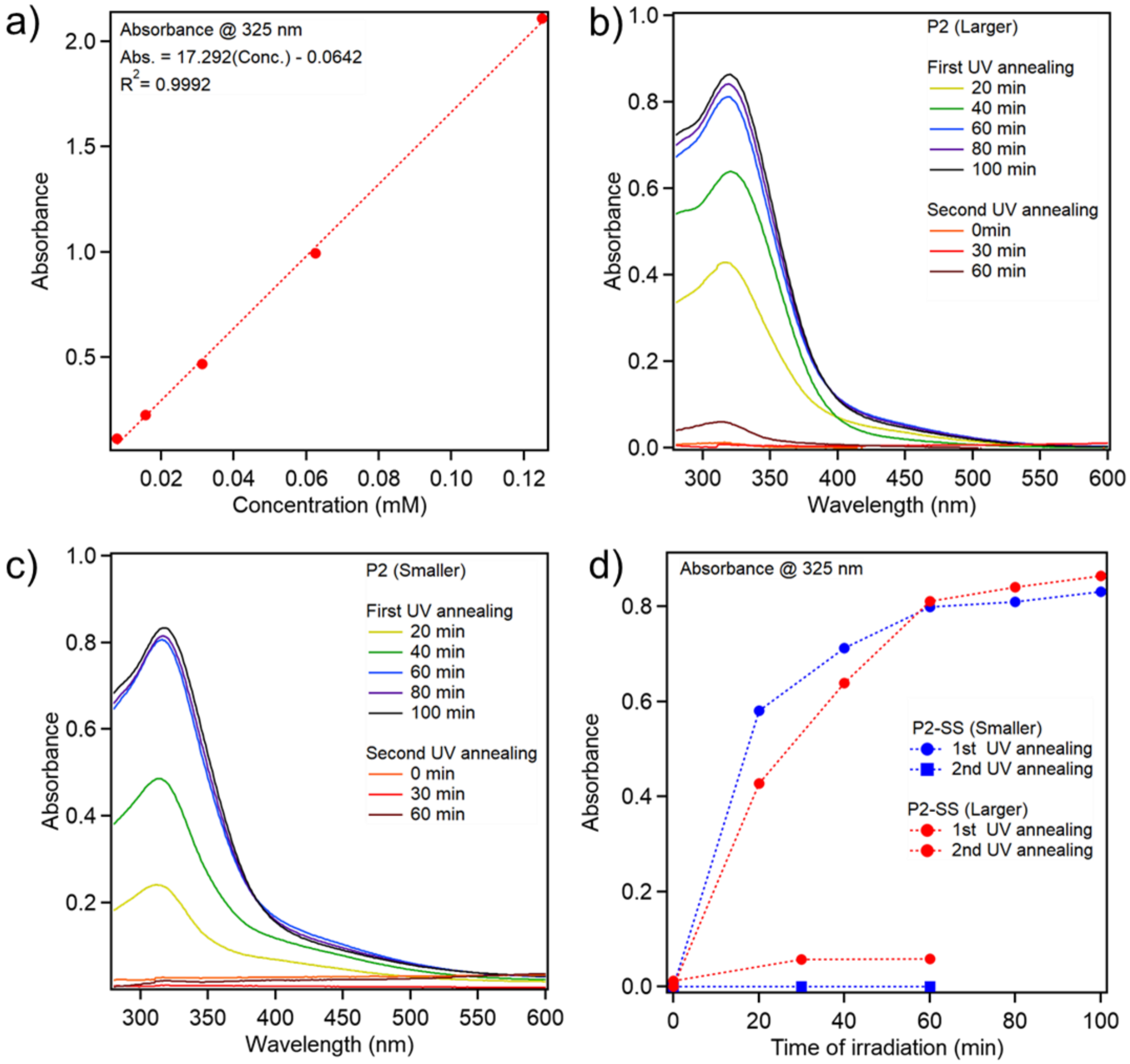

Figure S3. Quantification of **1-SS-1** small molecule released during UV annealing. **a)** UV-Vis calibration curve of **1-SS-1** in DMSO. **b)** UV-Vis measurements of **1-SS-1** in DMSO supernatant solution taken at various time points during UV annealing on **P2-SS** (larger) and **c)** (smaller). **d)** UV-Vis of supernatant solution to monitor the release of **1-SS-1** small molecule from **P2** into solution during UV.

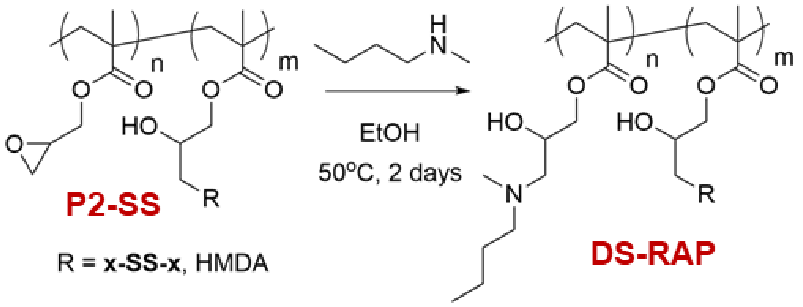

Figure S4. Reaction scheme for epoxy ring-opening in **P2-SS** particles using *N*-methylbutylamine to produce **DS-RAP**.

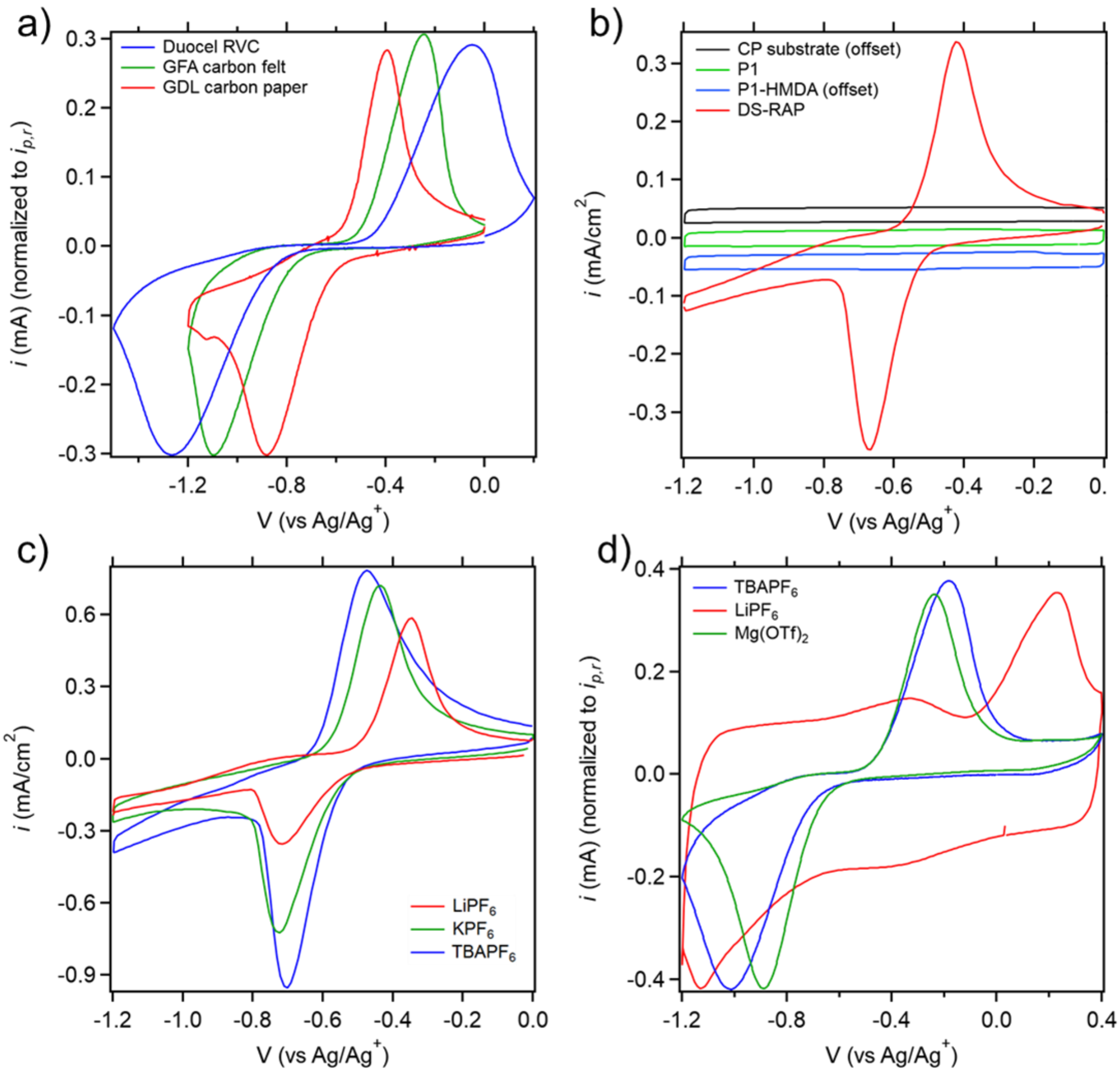

Figure S5. Cyclic voltammetry (CV) data for electrode selection and electrochemical reactivity. **a)** CV data for **2-SS-2** on different carbon electrodes. **b)** CVs for PGMA particles with or without **x-SS-x**, in multiple states of functionalization. CV profile for CP substrate and **P1-HMDA** were offset for clarity. **c)** CV data for larger **DS-RAP** using different electrolyte salts at 0.1 M concentration in ACN. **d)** CV data for larger DS-RAP using $TBAPF_6$, $LiPF_6$, or $Mg(OTf)_2$ at 0.1 M concentration in DMSO. All measurements were performed at 20 mV/s. Single cycle data is shown at cycle > 5 to allow for equilibration.

Electrode Selection. As shown in **Figure S5a**, the reactivity of the **1-SS-1** redox couple is electrode material-dependent.[3-5] Taking this into consideration, multiple electrode materials were tested: reticulated vitreous carbon (100 pores per inch, Duocel), carbon felt (Sigracell GFA 6 EA, SGL Carbon), and high temperature treated carbon fiber paper (Sigracet GDL 39 AA, SGL Carbon). Carbon paper (CP) was cut to 1 cm^2 pieces, while RVC and carbon felt electrodes were cut to 1×1×0.5 cm. All electrodes were mounted to Pt wire electrodes. CV traces for **Figure S5a**

were normalized to peak reduction current density (i_p) to aid visualization of the peak positions. Data is reported for cycle 5, allowing for stabilization of reduction product species concentration.

Electrolyte Selection for CV Measurements. Impact of electrolyte salt cation selection on **DS-RAP** was investigated using CV with 0.1 M salt in ACN (**Figure S5c**). The smaller alkali metal salts ($LiPF_6$, KPF_6) demonstrated reduced electrochemical reversibility as determined by the larger peak spacing compared to $TBAPF_6$. This is attributed to strong association of the metal cation to the reduced state thiolate anion, and resulting additional oxidative potential needed to re-oxidize the thiolate. The larger TBA^+ cation associates less strongly to the thiolate anion, resulting in easier recombination of neighboring thiolate anions to a disulfide during oxidation. A similar study was used for the selection of metallic cation salt in DMSO (**Figure S5d**). Using $LiPF_6$ results in large peak spacing compared to the $Mg(OTf)_2$. While reduction onset potentials remain consistent compared to those in ACN (ca. -0.6 V), the positive shift in the oxidation onset potential for $LiPF_6$ is exacerbated by the switch in solvents, also seen in $TBAPF_6$ in DMSO vs ACN (**Figure S5c**). CV traces for **Figure S5d** were normalized to peak reduction current density (i_p) to aid visualization of the peak positions.

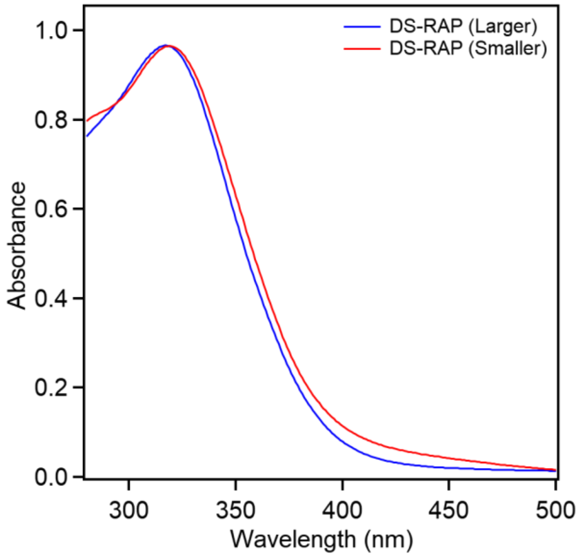

Figure S6. UV-Vis absorbance spectra for covalently-bound **x-SS-x** in **DS-RAP** dispersion.

Quantifying Level of Disulfide Crosslinker Functionalization. UV-Vis spectra was used to analyze the amount of functionalized disulfide crosslinkers via **x-SS-x** absorption for **DS-RAP** dispersions **(Figure S6)**. A low concentration of **DS-RAP** ($C_{particle}$ = 0.05 mg/mL) was required to avoid broadening resulting from scattering caused by large particle sizes.[6] Concentration of **x-SS-x** in **DS-RAP** (C_{DS}, mM, as determined by UV-Vis) multiplied by the molecular weight of **x-SS-x** (Mw = 264.26 g/mol) yields concentration of **x-SS-x** in dispersion as part of the particle (mg/mL). Dividing the concentration of **x-SS-x** by the concentration of **DS-RAP** particles yields the mass% of **x-SS-x** in **DS-RAP** (**Equation S1**).

Equation S1. $$mass\%_{x-SS-x} = \frac{C_{DS} \times 264.26\ g/mol}{C_{particle}}$$

The mass% of **x-SS-x** contained in **DS-RAP** was calculated to be 38% and 39% for smaller and larger **DS-RAP**, respectively, compared to a theoretical maximum of 48%. The mole percentage of **x-SS-x** contained in **DS-RAP** could be calculated as well. 2 wt% of HMDA is subtracted from the remained mass of PGMA. The mole% of **x-SS-x** contained in **DS-RAP** is 25% and 26% for smaller and large **DS-RAP**, respectively (33% theoretical maximum).

Calculation of Theoretical Specific Capacity. The theoretical capacity of a charge storage material in mAh/g is calculated by **Equation S2**.

Equation S2. $$Q_{theoretical} = \frac{nF}{3600 \times Mw_{x-SS-x}} \times mass\%_{x-SS-x}$$

The theoretical specific capacity of **1-SS-1** is 203 mAh/g. Taking the mass% of **x-SS-x** contained in **DS-RAP** as 38 and 39%, the theoretical capacity of **DS-RAP** is calculated to be 78 mAh/g and 79 mAh/g for smaller and larger **DS-RAP**, respectively.

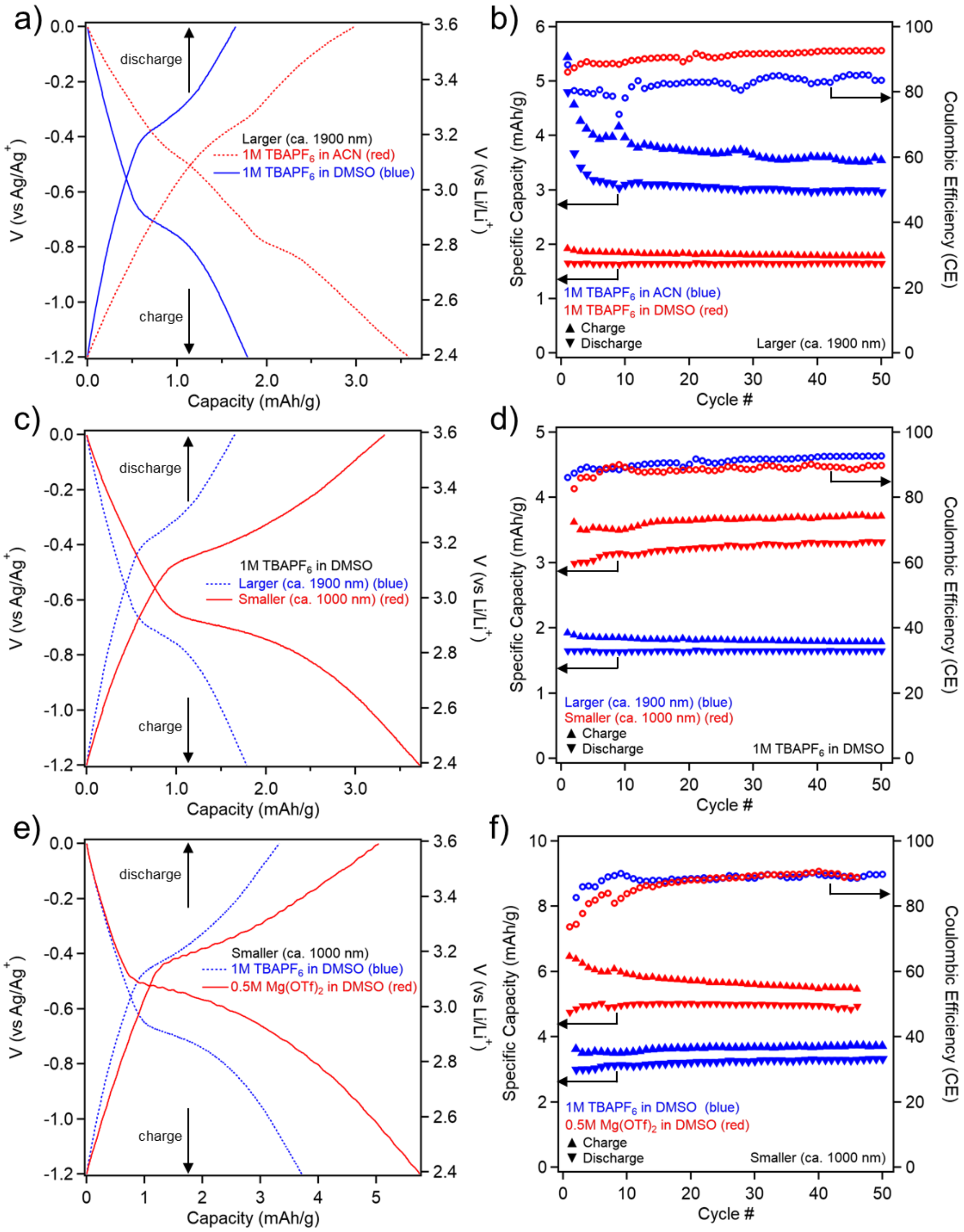

Figure S7. Galvanostatic cycling (GC) of **DS-RAP** using different cell preparations at a C-rate of 0.25C. Potential (V) vs Li/Li^+ is converted from Ag/Ag^+. **a)** Charge/discharge curves and **b)** charge/discharge specific capacity and Coulombic efficiency (CE) data for ca. 1900 nm **DS-RAP** with 1 M $TBAPF_6$ in ACN or DMSO, **c,d)** for ca. 1000 nm or ca. 1900 nm **DS-RAP** with 1 M $TBAPF_6$ in DMSO, and **e,f)** for ca. 1000 nm **DS-RAP** with 1 M $TBAPF_6$ or 0.5 M $Mg(OTf)_2$ in DMSO. All particle diameters are from measured values in their respective electrolyte (**Table S3**).

Table S4. Qualitative observations for screening of non-selected electrolyte solvents.

**Solvents screened**	**Observations**
**Acetonitrile**	Relatively low efficiency, GC curve suggests degradation
Propylene carbonate	Very low capacity, reductive degradation (observed in GC curve)
**Dimethyl sulfoxide**	Relatively low capacity, but high cycling stability and efficiency
Tetrahydrofuran	Very poor efficiency, suggestive of solvent degradation (via GC)
Ethylene carbonate/ dimethyl carbonate	Degradation at oxidative potentials approaching zero (via GC)

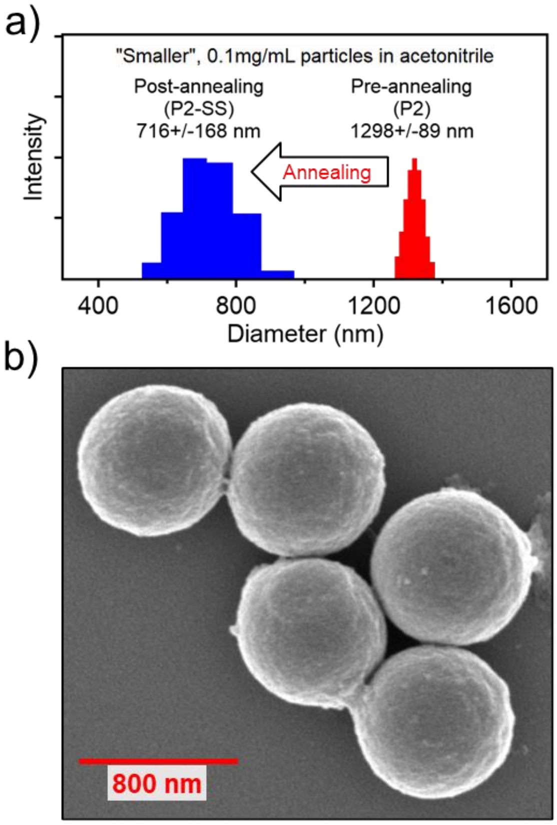

Figure S8. Particle size change characterization and scanning electron microscopy (SEM) imaging for smaller **DS-RAP**. **a)** Particle size reduction after photo-annealing as measured by dynamic light scattering (DLS) in acetonitrile (ACN) for smaller particles. **b)** SEM images of smaller **DS-RAP** particles.

Table S5. Summary **DS-RAP** specific discharge capacity (SDC) and Coulombic efficiency (CE) results under different conditions (0.25C C-rate)

Electrolyte	Diameter (nm)	C-rate	SDC (mAh/g)	Coulombic efficiency (%)
$TBAPF_6$/ ACN	1956 ± 45	0.25	2.97	83.7
$TBAPF_6$/ DMSO	1867 ± 46	0.25	1.65	92.7
$TBAPF_6$/ DMSO	1034 ± 57	0.25	3.32	89.5
$Mg(OTf)_2$/ DMSO	1013 ± 44	0.25	4.94	89.0
$Mg(OTf)_2$/ DMSO	1013 ± 44	0.10	15.21	Not stabilized

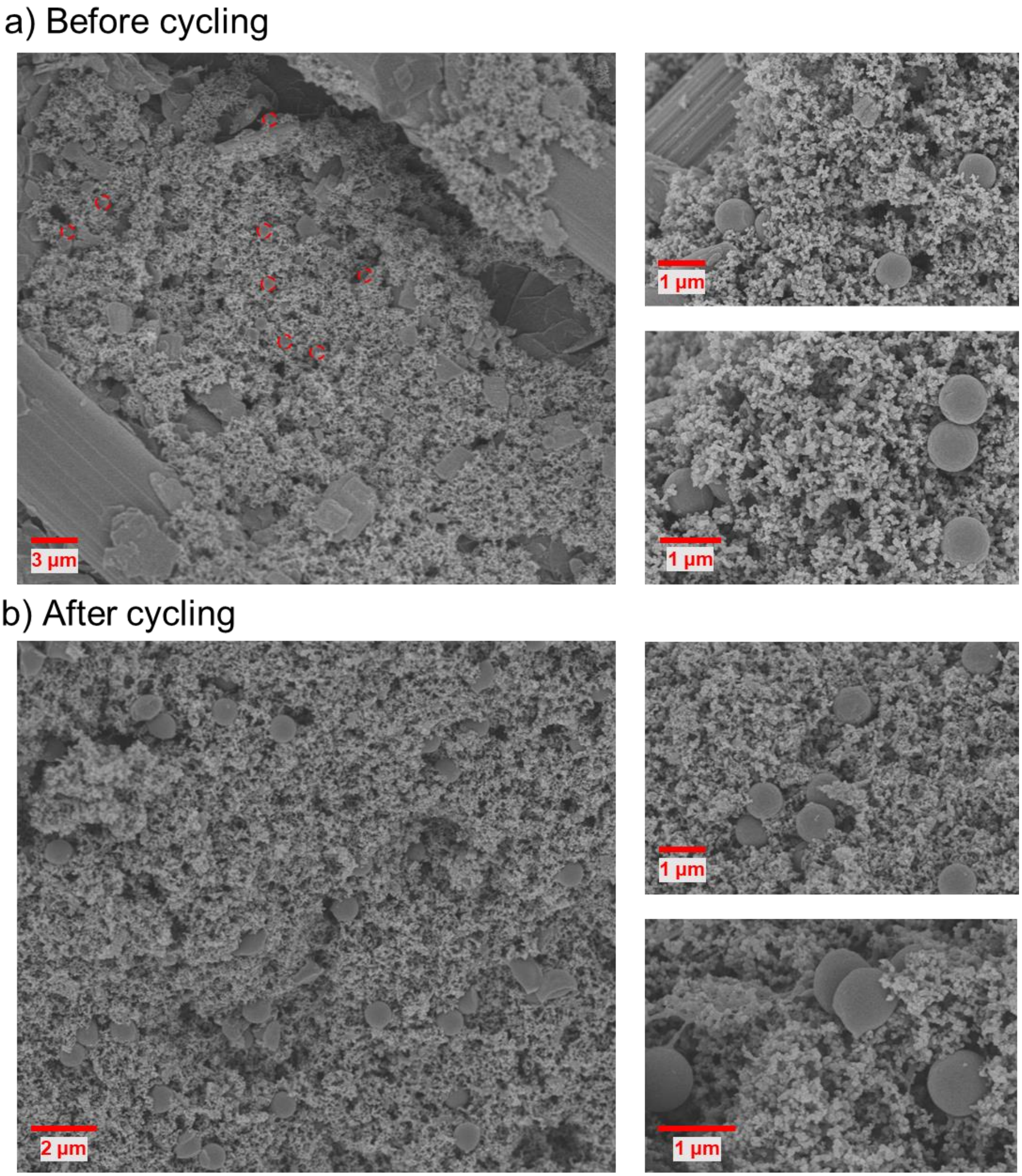

Figure S9. SEM images of smaller DS-RAP blended with carbon black before and after charge/discharge cycling. Red circles are added to the first low resolution image to aid in identifying several randomly selected particles. Post-cycling electrode sample was prepared by soaking twice in clean ACN prior to sputter coating. A complete removal of $Mg(OTf)_2$ supporting electrolyte was not validated prior to sputter coating.

References:

1. Viscosity of Acetonitrile – viscosity table and viscosity chart. Anton Paar Wiki. https://wiki.anton-paar.com/en/acetonitrile/

2. DMSO Physical Properties. Gaylord Chemical. https://www.gaylordchemical.com/literature/dmso-physical-properties

3. Pope, J. M.; Sato, T.; Shoji, E.; Oyama, N.; White, K. C.; Buttry, D. A. Organosulfur/Conducting Polymer Composite Cathodes. *J. Electrochem. Soc.* **2002**, *149* (7), A939. https://doi.org/10.1149/1.1482768.

4. Tatsuma, T.; Yokoyama, Y.; Buttry, D. A.; Oyama, N. Electrochemical Polymerization and Depolymerization of 2,5-Dimercapto-1,3,4-Thiadiazole. QCM and Spectroscopic Analysis. *J. Phys. Chem. B* **1997**, *101* (38), 7556–7562. https://doi.org/10.1021/jp971502a.

5. Pope, J. M.; Oyama, N. Organosulfur/Conducting Polymer Composite Cathodes: I. Voltammetric Study of the Polymerization and Depolymerization of 2,5-Dimercapto-1,3,4-thiadiazole in Acetonitrile. *J. Electrochem. Soc.* **1998**, *145* (6), 1893–1901. https://doi.org/10.1149/1.1838573.

6. van de Hulst, H. C. *Light scattering by small particles.* Courier Corporation. **1981**.
